# Cognitive Enhancement in Infants Associated with Increased Maternal Fruit Intake During Pregnancy: Results from a Birth Cohort Study with Validation in an Animal Model^☆^

**DOI:** 10.1016/j.ebiom.2016.04.025

**Published:** 2016-04-22

**Authors:** Francois V. Bolduc, Amanda Lau, Cory S. Rosenfelt, Steven Langer, Nan Wang, Lisa Smithson, Diana Lefebvre, R. Todd Alexander, Clayton T. Dickson, Liang Li, Allan B. Becker, Padmaja Subbarao, Stuart E. Turvey, Jacqueline Pei, Malcolm R. Sears, Piush J. Mandhane, The CHILD Study Investigators

**Affiliations:** a Department of Pediatrics, University of Alberta, Canada; b Department of Chemistry, University of Alberta, Canada; c Department of Medicine, McMaster University, Canada; d Department of Psychology, University of Alberta, Canada; e Manitoba Institute of Child Health, University of Manitoba, Canada; f Department of Pediatrics, University of Toronto, Canada; g Department of Pediatrics, University of British Columbia, Canada; h Department of Educational Psychology, University of Alberta, Canada; i Canadian Healthy Infant Longitudinal Development Study, Canada

**Keywords:** Gestational diet, Fruit, Birth cohort, *Drosophila* learning

## Abstract

In-utero nutrition is an under-studied aspect of cognitive development. Fruit has been an important dietary constituent for early hominins and humans. Among 808 eligible CHILD-Edmonton sub-cohort subjects, 688 (85%) had 1-year cognitive outcome data. We found that each maternal daily serving of fruit (sum of fruit plus 100% fruit juice) consumed during pregnancy was associated with a 2.38 point increase in 1-year cognitive development (95% CI 0.39, 4.37; p < 0.05). Consistent with this, we found 30% higher learning Performance index (PI) scores in *Drosophila* offspring from parents who consumed 30% fruit juice supplementation prenatally (PI: 85.7; SE 1.8; p < 0.05) compared to the offspring of standard diet parents (PI: 65.0 SE 3.4). Using the *Drosophila* model, we also show that the cyclic adenylate monophosphate (cAMP) pathway may be a major regulator of this effect, as prenatal fruit associated cognitive enhancement was blocked in *Drosophila rutabaga* mutants with reduced Ca^2 +^-Calmodulin-dependent adenylyl cyclase. Moreover, gestation is a critical time for this effect as postnatal fruit intake did not enhance cognitive performance in either humans or *Drosophila*. Our study supports increased fruit consumption during pregnancy with significant increases in infant cognitive performance. Validation in *Drosophila* helps control for potential participant bias or unmeasured confounders.

## Highlights

• Gestational fruit intake positively correlates with infant cognitive performance.
• Similar findings in a birth cohort and in *Drosophila* learning and memory scores
• Cyclic adenylate monophosphate (cAMP) pathway may be a major regulator of this effect.
• Postnatal fruit intake did not enhance cognitive outcomes in humans or *Drosophila*.

Fruits have been an important part of the human diet for thousands of years. We wanted to know if more fruit intake improves our ability to learn. Using data from the Canadian Healthy Infant Longitudinal Development (CHILD) study, we found that mothers who ate more fruit during pregnancy had children who did better on developmental testing at 1 year of age. Similarly, fruit flies had improved learning and memory if their parents had more fruit juice in their diet. In both humans and in the flies, there was no improvement in learning when only the babies were fed fruit.

## Graphical Abstract

**Figure:**
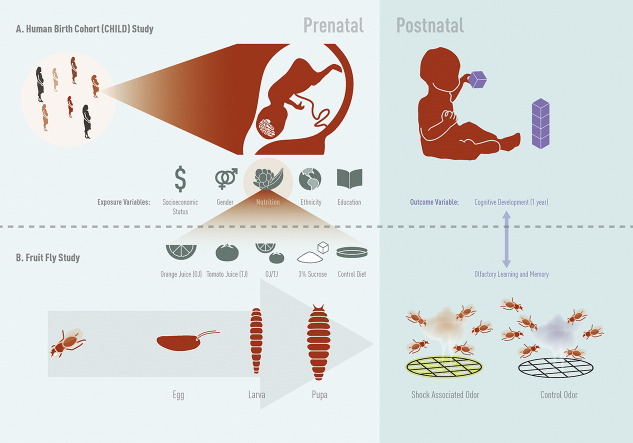

## 1 Background

Cognitive performance is determined by genetic and environmental factors (Tucker-Drob and Briley, 2014). In-utero nutrition is an under-studied aspect of cognitive development (Godfrey and Barker, 2000). Whereas supplemental prenatal folic acid prevents neural tube defects (Gomes et al., 2015), other nutrients required for optimal cognitive development remain largely unknown. Fruits, foliage, nuts, and seeds have been traditionally important dietary constituents for humans and early hominins (Ungar and Sponheimer, 2011). There is renewed interest in examining whole food impact on health outcomes rather than macro- and micro-nutrient separately (Simpson et al., 2015).

We used a combination of human epidemiological studies and an in vivo model organism to investigate the influence of prenatal and postnatal fruit consumption on early cognitive performance.

The Canadian Healthy Infant Longitudinal Development (CHILD) study is a general population-representative birth cohort of term and near-term infants recruited while mothers were pregnant (Subbarao et al., 2015). Although epidemiological studies can infer associations, direct experimental studies, especially in model species, provide more causal and generalizable inferences. The *Drosophila* olfactory learning and memory model (Bolduc and Tully, 2009) has been used extensively to examine intellectual disability (van der Voet et al., 2014). Additionally, the *Drosophila* diet is simple and primarily composed of protein and carbohydrate (Simpson et al., 2015) allowing for easy dietary manipulation to recapitulate human findings. For example, *Drosophila* larvae were more viable and developed faster with increasing dietary folic acid (Blatch et al., 2010).

The cyclic adenylate monophosphate (cAMP) and cAMP response element binding protein (CREB) pathway was among the first pathways linked to learning and memory in Aplysia and Drosophila (Yin et al., 1994, Brunelli et al., 1976). It was subsequently determined that Fragile X syndrome patients have altered cAMP signaling (Kelley et al., 2007) while Rubinstein-Taybi syndrome patients have a CREB pathway mutation (Petrij et al., 1995). Flies have since became a model used extensively by several groups to understand the molecular basis of intellectual disability (Androschuk et al., 2015). *Drosophila* mutants of *rutabaga* (Levin et al., 1992), a gene that encodes for a calmodulin dependent adenylate cyclase that converts ATP to cAMP (Kandel and Schwartz, 1982), were among the first learning mutants isolated in a forward genetic screen for learning.

CHILD Edmonton sub-cohort data were analyzed to ascertain the effect of prenatal and postnatal fruit intake on 1-year neurodevelopment. Higher gestational fruit intake was associated with enhanced cognitive performance at 1 year of age. We then took advantage of the *Drosophila* olfactory learning and memory model (Bolduc and Tully, 2009) to ascertain whether cognitive enhancement in healthy individuals following high prenatal fruit intake persisted across species and to decipher the molecular mechanisms with a focus on cAMP.

## 2 Methods

### 2.1 CHILD

Expectant mothers (any gestational age (GA) prior to delivery) giving birth in Edmonton, Canada were invited to participate at the CHILD Edmonton site (Subbarao et al., 2015). Inclusion and exclusion criteria are in Supplemental Table e1. A study overview with measurements at each age is in Supplemental Table e2. Parents were seen at recruitment, generally in the second or third trimester. Children were seen within 1 month of the child's 1st birthday during the day at a time that the parent felt the child would be most awake (i.e. not during nap time). Mothers provided consent both for themselves and their child. Father's consent was not necessary for the child to participate. The University of Alberta Health Research Ethics Board approved the CHILD study (Pro00002099).

Cognitive development was assessed by the Bayley scale of infant development (BSID-III); a well-validated instrument of cognitive development for infants 1 to 42 months of age. The BSID-III cognitive scale (91-items) assesses visual preference, attention, memory, exploration, manipulation, and concept formation. The infant adaptive behavior questionnaire (241-items) assesses communication use, self-care, self-direction, health and safety, leisure, and social behavior. The BSID-III was completed and scored by trained research assistants. A food frequency questionnaire (FFQ) developed by the Fred Hutchinson Cancer Research Center was modified to reflect Canadian multi-ethnic food choices. The 175-item self-administered FFQ was completed at enrolment. It asked pregnant mothers to report the frequency and portion size of food since becoming pregnant. Total fruit intake (“5-a-day” method) was the sum of “servings of fruit, not including juices” plus “servings of juice” per day (Kristal et al., 2000). The food frequency questionnaire defined a medium serving of fruit as either a ¾ cup of 100% fruit juice, ½ cup of fresh fruit, or ¼ cup of dried fruit. The University of Minnesota Nutrition Data Systems for Research (Dennis et al., 1980) was “Canadianized” to include foods from multiple ethnic backgrounds and used to calculate the nutrient intake (e.g. lycopene and fructose) for each subject. After questionnaire completion (bubble forms), record data entry was completed by Hutchinson Cancer Center nutrition coordinating center (NCC) staff who are trained and certified in dietary data entry. The NCC requested additional information from the CHILD study if questionable items (e.g. extreme values) or data quality issues (e.g. missing) were identified. In addition, the CHILD Edmonton reviewed the raw FFQ for fruit intake values > 6 servings per day (n = 38). The analysis of the FFQ for nutrient consumption was completed by the NCC using the University of Minnesota Nutrition Data Systems for Research (NDSR) software (Dennis et al., 1980). The Healthy Eating Index (2010) (Guenther et al., 2013) was calculated for each participant. Factors associated with neurodevelopment such as the GA at which the baby was born, maternal education, gestational diabetes (GDM), and socioeconomic status (SES) were assessed longitudinally throughout the study. Parent-reported SES was assessed by family income (Chen et al., 2004), maternal education, and subjectively using the MacArthur Scale of Subjective Social Status (Adler et al., 2000, Goodman et al., 2001).

### 2.2 *Drosophila* Model of Learning and Memory

#### 2.2.1 *Drosophila* Stocks

**Fig. 2:**
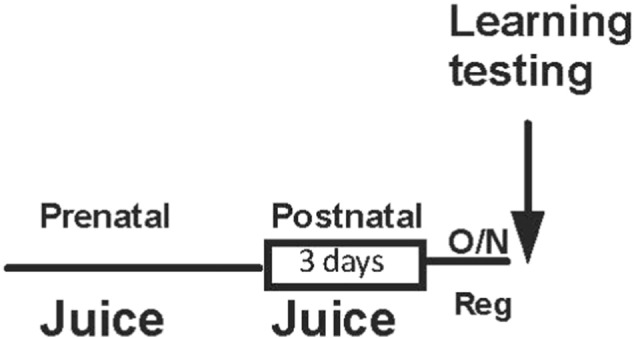
Schematic representation of prenatal feeding fruit juice supplemented diet in *Drosophila*.

Wild-type *Drosophila* with w118 canton-s background were raised at 22 °C and 50% humidity with 12 h light:dark cycle. Drosophila adenylate cyclase *Rutabaga* (Levin et al., 1992, Kandel and Schwartz, 1982) mutant is characterized by its lack of cAMP production and learning defects. The basal performance of rutabaga mutants has previously been shown to be lower than wild type *Drosophila* when raised on regular diet (Livingstone et al., 1984). *Drosophila* and *Drosophila rutabaga* (Levin et al., 1992) were raised on the standard Cold Spring Harbor Lab diet or on food supplemented with 30% (volume for volume) orange juice (OJ; a source of fructose but low in lycopene; Minute Maid tetrapak), 30% (volume for volume) tomato juice (TJ; a source of lycopene; Heinz tetrapak), or 15% OJ and 15% TJ. *Drosophila* were transferred to regular food for both developmental and post-natal feeding experiments the night before training (Fig. 2).

#### 2.2.2 Olfactory Learning and Memory Testing

*Drosophila* can learn the association between a footshock and an odor after a single training session. For learning testing, 100 flies, 3 days old, of the same genotype are loaded in the training apparatus. Flies are presented with a first odor (Octanol-OCT) at the same time as footshocks are delivered through an electrified grid for 30 s. The flies are then given time to rest before being presented with a second different odor (Methylcyclohexanol-MCH) for 30 s but this time without shock. Flies are then brought to a choice point where they can go to tubes containing either the first or second odor. The flies are then trapped and the number of flies on each side enumerated. The test is then repeated with the shock being paired with the second odor. For long-term memory (LTM), tested typically at 1-day after spaced training, flies are trained for 3 cycles with 15 min rest interval between each training session. Flies are placed on regular food for 24 h before being tested for their recall of the odor to be avoided the next day. Flies are tested using sensory controls for their ability to smell the odors and feel the shock to confirm the effect observed is related to learning or memory (Bolduc et al., 2008).

### 2.3 Statistical Analysis

Children with known causes of developmental delay (e.g. Down syndrome, autism spectrum disorder) were excluded from analysis. BSID-III raw scores were transformed into a composite score (a mean of 100 and a SD of 15) using the BSID-III scoring rubric. Univariate analysis (*t*-test, ANOVA, and Pearson correlations) was used to identify significant (p < 0.05) exposures associated with cognitive development at 1 year of age (primary outcome). Significant factors from the univariate analyses were included in multivariate linear regression. Interactions were included if they were significant or changed the main effect beta-coefficients by 10% or more. The GA at which the mother completed the FFQ was included in the analysis. We only tested interactions if they were shown to be previously associated (e.g. gestational age and gestational diabetes) or had potential to increase explanatory capacity (e.g. gestational age and gestational age at which the food frequency questionnaire was completed). A separate backward selection multivariate regression was used to identify what nutrient constituents are associated with cognitive development. Similar analyses were used for the adaptive development (secondary outcome). We did not correct for multiple-outcomes for the adaptive development. Statistical analyses were completed using STATA 13.1 (STATA corp.).

A One-Way ANOVA followed by a Tukey post-test was performed for the *Drosophila* experiments where multiple groups are present and a *t-*test where only 2 groups were present. Statistical analysis was performed with Prism and JMP.

### 2.4 Financial Support

The Canadian Institutes of Health Research (CIHR) and the Allergy, Genes and Environment (AllerGen) Network of Centres of Excellence provided core support for CHILD. CIHR and the Women and Children's Health Research Institute (WCHRI) at the University of Alberta specifically funded this research.

## 3 Results

Of the 841 participants recruited to the CHILD study in Edmonton, Alberta, Canada, 33 infants did not meet inclusion criteria resulting in an eligible sample of 808 children. Over these, 688 (85%) of CHILD subjects had cognitive outcome data (population standardized mean of 100 points with a standard deviation (SD) of 15), assessed by the Bayley Scale of Infant Development (BSID-III), at 1 year of age. There was no significance between those with and without cognitive outcomes for gender, first-born, parents' marital status, or smoke exposure in the home. Individuals with cognitive outcomes at age 1 were predominantly Caucasian (467/668; 69.9%, Table 1) compared to 54.7% of subjects missing cognitive outcomes at 1 year of age (58/106; p = 0.002). Individuals with BSID-III data at one year had mothers with a mean age of 31.6 years (95% CI 31.2, 31.9) versus 29.8 years (95% CI 28.9, 30.6); p < 0.001) for those subjects without cognitive outcomes at 1 year of age. Individuals with outcomes at age 1 were more likely to have a family income of $60,000 or over (86.5%; 572/661; Table 1) compared to 73.6% subjects without cognitive outcomes at 1 year of age (67/91; p = 0.001). Similarly, individuals with cognitive outcomes at age 1 were more likely to have a mother attend post-secondary education (92.6%; 613/662) compared to 81.4% of subjects without cognitive outcomes at 1 year of age (83/102; p < 0.001). The mean breastfeeding duration was 8.3 months (95% CI 8.0, 8.6) for participants with cognitive outcome data versus 9.3 months (95% CI 8.5, 10; p < 0.05) for those without outcomes at 1 year of age. Infants of mothers with cognitive outcomes at 1 year of age had a higher healthy eating index (Guenther et al., 2013) (HEI mean 77.30; 95% CI 72.7, 73.9; n = 607) compared than those infants missing cognitive outcomes at 1 year of age (mean 71.0; 95% CI 68.9, 73.2; n = 89; p = 0.02; Table 1). The mean maternal calorie intake during pregnancy was 2093 cal per day (95% CI: 2015, 2151; n = 605). Infants of mothers with cognitive outcomes at 1 year of age had 2.95 servings of fruit per day (95% CI 2.8, 3.1; Table 1) (calculated as the sum of “servings of fruit, not including juices” plus “servings of juice” per day (Kristal et al., 2000)) compared to 3.5 serving of fruit per day for mother of infants without cognitive outcomes at 1 year of age (95% CI 3.1, 4.0; p = 0.01). The largest proportion of women (26%) consumed < 1.5 servings of fruit per day and 2/3 of women had < 3.5 servings of fruit per day. For each daily serving of fruit the mother consumed during pregnancy, there was an 11% (OR 1.11, 95% CI 1.01, 1.21; p = 0.03) increased chance of the infant having fruit at 6 months of age.

### 3.1 Children of Mothers ith Higher Gestational Maternal Fruit Intake Demonstrated Enhanced Cognitive Performance

**Fig. 3:**
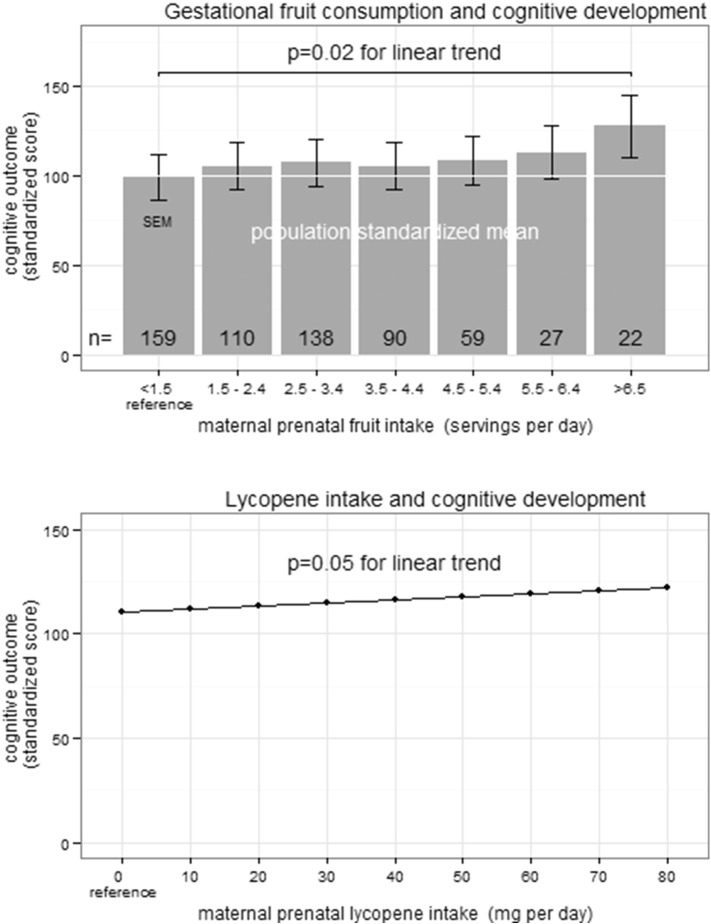
Increased infant cognitive development at 1 year of age as a result of increased maternal prenatal fruit consumption. A) The standardized population mean is 100 (SD 15). In multivariate analysis, cognitive performance of infants at 1 year of age was increased 2.38 points (95% CI 0.39–4.37; p = 0.02) for each daily serving of fruit the mother consumed during pregnancy. B) In multivariate analysis, cognitive development of infants at 1 year of age was increased 0.14 points (95% CI 0.00, 0.28; p = 0.05) for each 1 mg increase in maternal lycopene consumption during pregnancy.

Increased daily fruit intake during pregnancy was significantly associated with increased 1-year cognitive development (Supplemental Table e3) and adaptive development in univariate analyses (Supplemental Table e4). In multivariate analysis, each daily serving of fruit consumed during pregnancy was associated with a 2.38 point increase in cognitive development at 1 year of age (95% CI 0.39, 4.37; p < 0.05; Fig. 3 and Table 2) when controlling for SES, maternal education, GA, maternal gestational HEI, GDM, and maternal vitamin supplementation. We have completed a sensitivity analysis where we included the HEI, total calorie count, total dietary fiber, and total omega 3 fatty acid intake in the multivariate analysis. None of these were significant for predicting cognitive development or adaptive development at 1 year of age. The MacArthur Scale of Subjective Social Status was also not significant in univariate or in multivariate analyses. There was no significant interaction between fruit consumption during pregnancy and either gestational diabetes or measures of socioeconomic status (maternal education or family income). The impact of prenatal fruit intake on cognitive development was greatest among children born earlier in gestation. For each week the infant was born closer to 34.3 weeks GA (reference), there was a 0.36 point (95% CI 0.00, 0.73; p = 0.06) increase in cognitive development for each serving of fruit. When fruit was categorized into a dichotomous predictor above and below 7 servings of fruit daily, 7 or more servings of fruit per diem was associated with a 5.10 point increase (1/3 of the population SD) in cognitive development (95% CI 0.65, 9.55; p < 0.05). When analyzing for specific nutrients rather than fruit, we observed a 0.14 point increase in cognitive development for each 1 mg increase in lycopene consumed per day during pregnancy (95% CI 0.00, 0.28; p = 0.05; Fig. 3B).

**Fig. 4:**
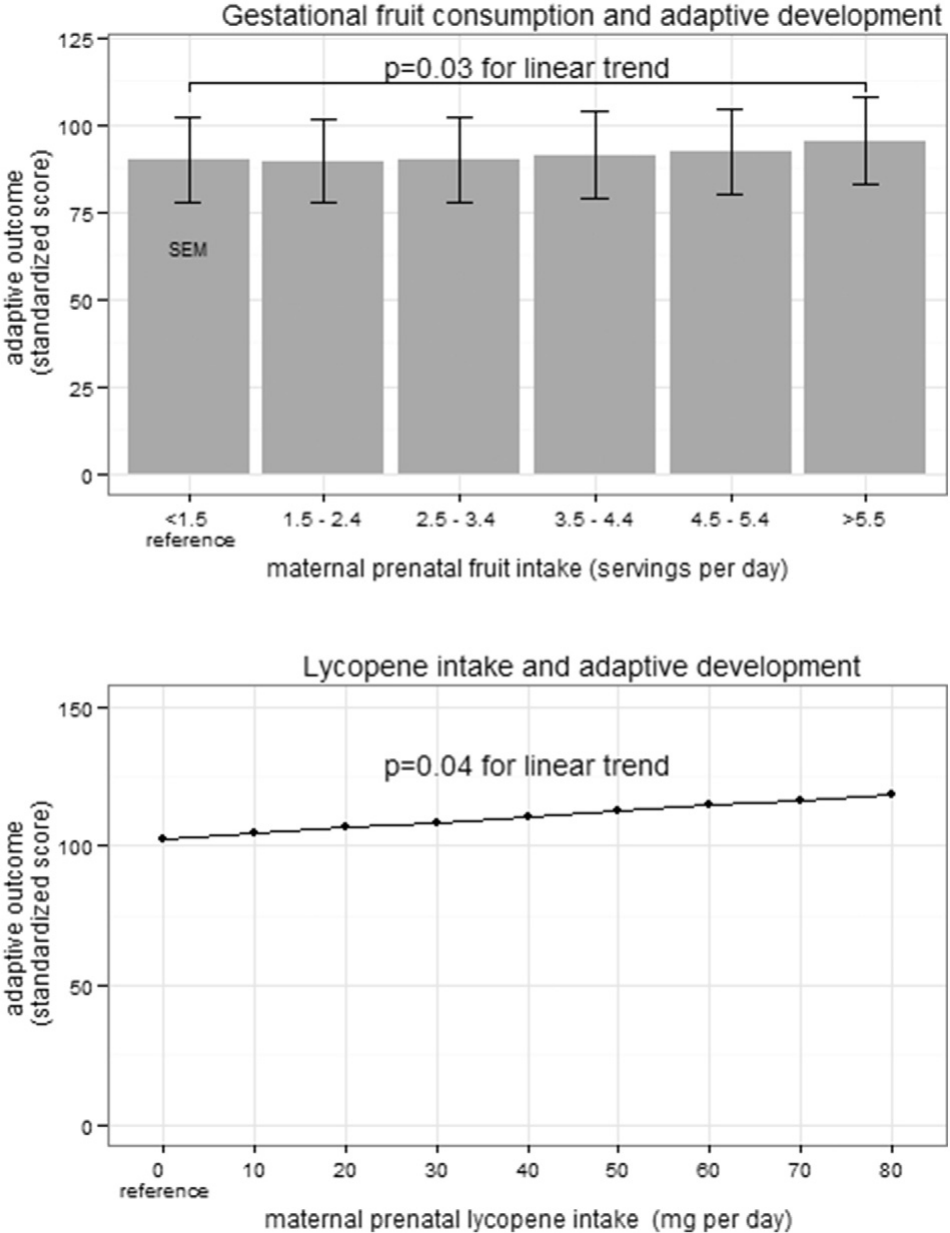
Increased infant adaptive development at 1 year of age as a result of increased maternal prenatal fruit and lycopene consumption. The standardized population mean is 100 (SD 15) A) In multivariate analysis, adaptive development of infants at 1 year of age was increased 0.67 points (95% CI 0.07–1.27; p = 0.03) for each daily serving of fruit the mother consumed during pregnancy. B) In multivariate analysis, adaptive development of infants at 1 year of age was increased 0.2 points (95% CI 0.01–0.39; p = 0.04) for each 1 mg increase in maternal lycopene consumption during pregnancy.

Each serving of fruit consumed during pregnancy was also associated with a 0.67 point increase in 1-year adaptive development (95% CI 0.07–1.27; p < 0.05; Fig. 4A and Table 3). There was a 0.21 point increase in adaptive development for each 1 mg increase in gestational daily lycopene intake (95% CI 0.01–0.39; p < 0.05; Fig. 4B). Fructose intake during pregnancy was associated with a 0.06 point increase in 1-year adaptive development for each 1 mg increase (95% CI 0.00, 0.13; p = 0.05; Supplemental Table 3). Post-natal fruit intake at 6 months of age (presence or absence) was not associated with enhanced cognitive development (univariate and multivariate analyses) but was associated with improved adaptive development, although the impact would be minimal clinically (2.66 point increase; 95% CI 0.28 to 5.03; p = 0.03).

### 3.2 Prenatal Fruit Juice Supplementation Enhances *Drosophila* Offspring Olfactory-associated Learning and Memory

**Fig. 5:**
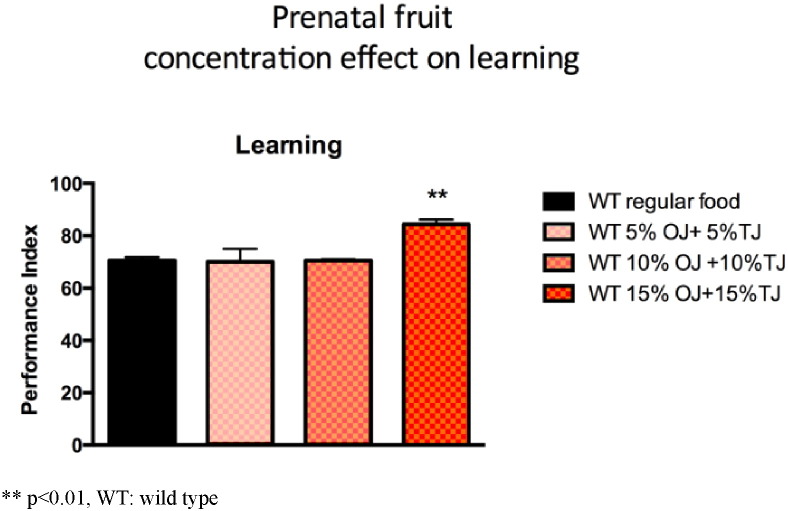
Performance during olfactory learning of wild-type Drosophila fed a regular diet or a diet supplemented with a combination of orange and tomato juice (OJ + TJ). 15% orange juice combined with 15% tomato juice is associated with significantly higher learning scores. (N = 2–6 per group; p = 0.0048 ANOVA). Error bars depict ± s.e.m, **p < 0.01, WT: wild type.

We performed a dose response curve and observed significantly higher Performance index (PI) learning scores in the offspring of wild-type *Drosophila* supplemented with 15% OJ and 15% TJ (30% total concentration) when compared to the offspring of wild-type flies whose parents were on standard diet alone (OJ + TJ PI: 85.7; SE 1.8 versus Regular PI: 65.0 SE 3.4; p < 0.05; Fig. 5). There was no significant learning effect observed with 10% and 20% total concentration (equal parts) of OJ + TJ.

**Fig. 6:**
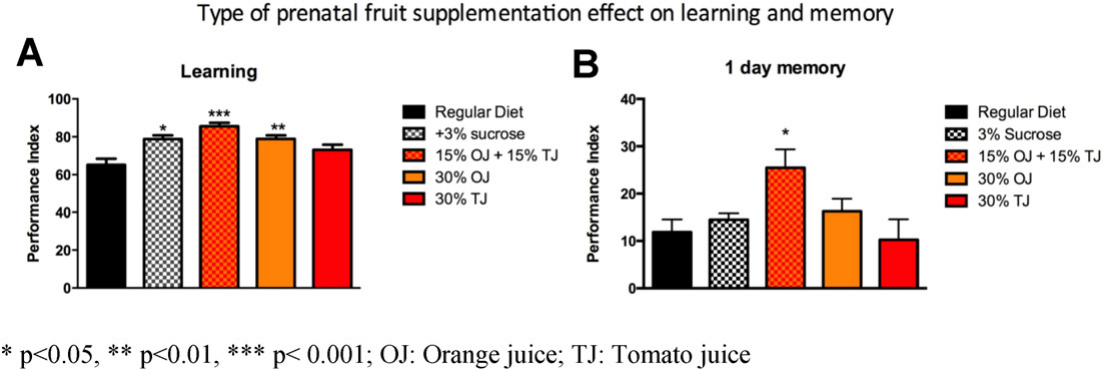
A) Wild-type flies fed prenatally with orange juice (OJ) alone (N = 6; p = 0.0048; ANOVA), sucrose alone (N = 4; p = 0.0149), or the combination of orange and tomato juice (TJ) (N = 6; p = 0.0003) display a significant increase in learning performance compared to flies fed a regular diet, whereas flies fed tomato juice alone do not demonstrate significant learning enhancement. B) Long-term memory at 1 day after spaced training is significantly enhanced in flies fed prenatally the orange and tomato juice mixture (N = 8; p = 0.0117, ANOVA). There is no effect of sucrose, orange or tomato juice feeding. Error bars depict ± s.e.m, *p < 0.05, **p < 0.01, ***p < 0.001; OJ: Orange juice; TJ: Tomato juice.

In addition to 30% OJ + TJ, we also observed higher learning PI scores among offspring of mothers supplemented with 30% OJ alone (OJ PI: 79; SE 1; p < 0.05 versus regular) and 3% sucrose alone (PI: 78.6 SE 1.9; p < 0.05; Fig. 6A) compared to the offspring of wild-type *Drosophila* whose mothers were on standard diet alone. TJ alone (30%) did not result in enhanced learning. The effect of fruit on learning was not due to changes in fly olfaction or sensation (Supplementary Fig. e1). Due to the transient nature of memory formed after a single training session in human and *Drosophila*, we sought to determine if long-term memory (LTM) was also influenced by prenatal fruit supplementation. We tested the effect of a similar diet modification on 1 day-memory after spaced training, which corresponds to LTM in *Drosophila*. For LTM, only the 30% OJ + TJ combination enhanced memory (PI: 25.6 SE 3.8; p < 0.05) compared to the regular diet controls (PI: 11.8 SE 2.7; Fig. 6B). Neither 30% OJ (PI: 16.3 SE 2.7), 3% sucrose (PI: 14.5 SE 1.1) nor 30% TJ (PI: 10.4 SE 4.4) had a significant effect on memory.

**Fig. 7:**
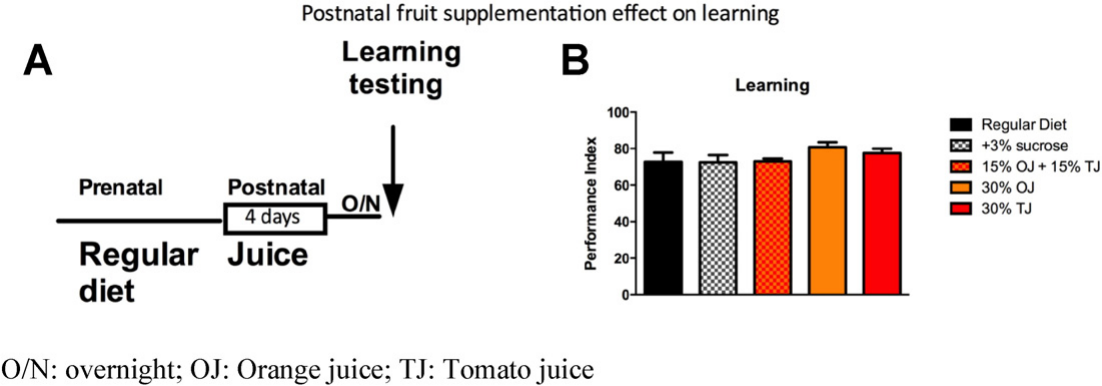
A) Schematic of adult fruit supplementation. B) Fruit supplementation to adult flies for 4 days does not cause significant learning improvement. Error bars depict ± s.e.m., O/N: overnight; OJ: Orange juice; TJ: Tomato juice.

Importantly, similar to the human cohort, learning enhancement was not seen when flies were fed the same fruit supplemented diet after birth for 4 days prior to testing (Fig. 7A and B).

**Fig. 8:**
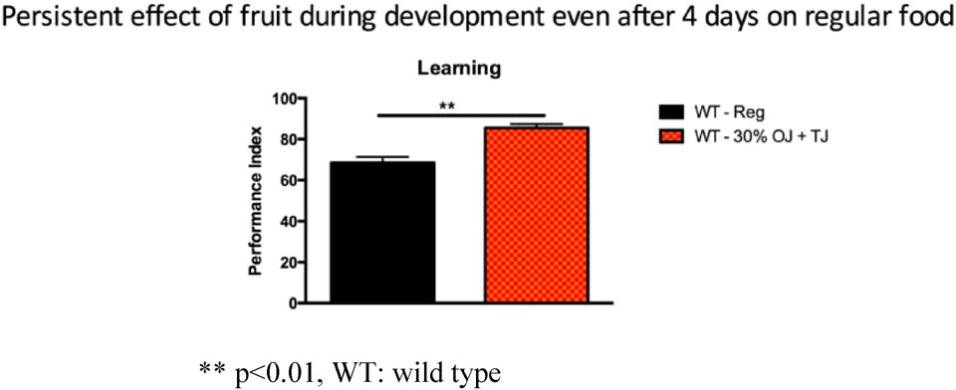
Wild-type flies fed prenatally with 30% (equal volumes) orange and tomato juice but on a regular diet for 4 days postnatal showed a persistent increase in learning (N = 4; Performance index = 85.5; SEM 0.9) compared to wild-type flies on a regular pre- and post-natal diet (N = 4; Performance index = 68.5; SEM 2; p = 0.0027). Error bars depict ± s.e.m, **p < 0.01, WT: wild type.

The observed enhanced learning was persistent over time. Wild-type flies, fed prenatally with 30% OJ + TJ, on a regular diet for 4 days after birth continued to show enhanced learning (p < 0.005) compared to wild-type flies on a regular diet (Fig. 8).

### 3.3 The cAMP Pathway is Central to Cognitive Enhancement by Prenatal Fruit Juice Supplementation

**Fig. 9:**
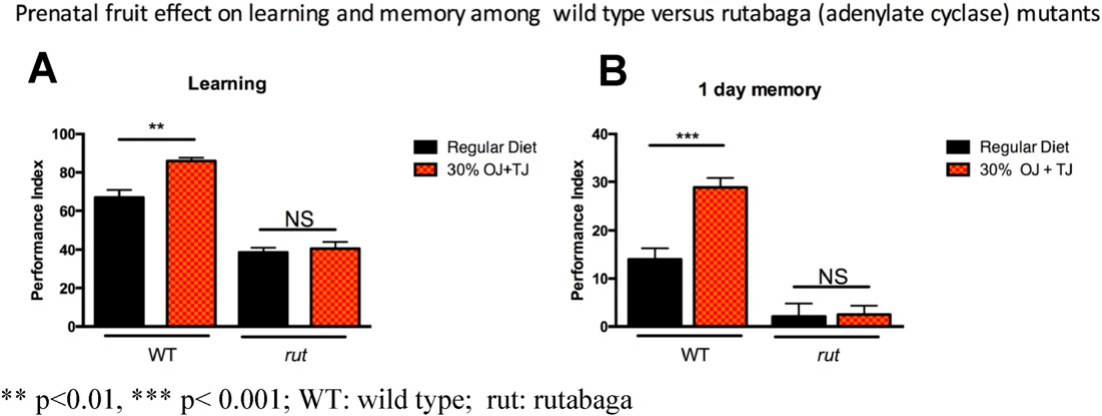
Olfactory learning and memory enhancement following prenatal fruit diet supplementation is blocked completely in the *Drosophila* adenylate cyclase mutant, *rutabaga*. A) As seen before learning is enhanced in wild-type flies fed on prenatal fruit supplemented diet (N = 6, p = 0.0015) whereas no enhancement is seen in *rutabaga* mutant flies. B) Similarly, 1 day memory after spaced training is enhanced in wild-type flies from the prenatal fruit diet group (N = 8, p = 0.0002) while rutabaga raised on the same diet did not show any memory enhancement. Error bars depict ± s.e.m, **p < 0.01, *** p < 0.001; WT: wild type; rut: rutabaga.

*Rutabaga* mutants submitted to 30% OJ + TJ supplementation failed to show any significant enhancement of learning (PI: 38.5 SE: 2.3) (Fig. 9A) or memory (PI: 2.12 SE: 2.5) (Fig. 9B) compared to wild-type *Drosophila*. Sensory controls for odor and shock did not show significant defects in *D. rutabaga* (Supplementary Fig. e1) (Bolduc et al., 2008).

## 4 Discussion

We have demonstrated that increasing prenatal fruit intake is associated with increased cognitive development in children using data from a population-based cohort. The influence of fruit was slightly greater among infants born earlier in gestation. Lycopene, a red carotene with antioxidant properties, was the food nutrient most strongly associated with cognitive development. Using the olfactory classical conditioning model of *Drosophila*, we explored the idea that a prenatal fruit-enhanced diet produces progeny with superior cognitive (learning and memory) abilities. Not only was this evident, but we also showed that this enhancement appears to require adenylate cyclase function as enhancement was not observed in *D. rutabaga* genetic mutants. The result of this study strongly supports the daily consumption of fruit as part of the prenatal diet for the general population of pregnant women.

The cAMP pathway has been implicated in physiological and pathological learning, but cognitive enhancement in healthy individuals has not been previously observed. In *Drosophila,* cAMP signaling during development is required for fine-tuning mushroom-body output neurons, a key component of learning and memory (Hige et al., 2015). Similarly, reducing cAMP degradation postnatally (inhibiting cAMP phosphodiesterase) in wild-type *Drosophila* did not enhance learning and memory (Choi et al., 2015). The cognitive effect observed in this study, mediated through cAMP, appears to be dependent on developmental changes occurring in the brain that cannot be recapitulated by post-natal supplementation. However, our study cannot determine if activation of the cAMP pathway alone during gestation is sufficient to enhance learning. Further research is required to identify the signaling pathways, funneling through cAMP, that are affected by the fruit-derived diet.

Fruit may improve learning and memory through improved antioxidant status. Increased antioxidant consumption is associated with improved learning and memory in older adults (Polidori et al., 2009, Peneau et al., 2011, Galli et al., 2002, Hritcu et al., 2014, Joseph et al., 1999) and antioxidant deficiency with adverse pregnancy outcomes. Lycopene deficiency is associated with preterm labor and intrauterine growth restriction (Sharma et al., 2003). A study of infants who died during the first year of life found that preterm infants had lower carotenoid levels in the frontal cortex and hippocampus, compared with term infants (Vishwanathan et al., 2014). Future *Drosophila* model studies will allow us to test the hypothesis that increased prenatal antioxidant status improves offspring learning and memory.

There are several strengths associated with this study. The relatively large sample size with a rich CHILD dataset allows us to control for confounders associated with fruit consumption and neurodevelopment. Pre-natal nutrition was prospectively collected reducing recall bias and 1-year cognitive development was objectively assessed using the current gold standard. While the CHILD sample is somewhat biased towards higher SES and advanced maternal education, the use of an approach combining human and *Drosophila* investigations helps address concerns around sample generalizability and potential bias due to unmeasured confounders.

Cognitive development at 1 year has a low correlation with cognitive development at 3 years (Aylward, 2004, Hack et al., 2005). Future studies are needed to examine the long-term impact of gestational fruit consumption. There is some evidence to suggest that the impact of fruit may be long lasting. We observed that the enhanced fruit-associated learning in *Drosophila* was persistent with increasing age. The Western Australian Pregnancy Cohort (RAINE) reported that increased fruit consumption at 1 year was positively associated with cognitive development at 10 years (Nyaradi et al., 2013). We did not find that post-natal fruit supplementation influenced learning in humans or the *Drosophila* model. The RAINE study did not control for gestational fruit intake that may have confounded their results; we found that increased prenatal fruit consumption was associated with increased odds of the infant being fed fruit at 6 months of age (supplemental results).

CHILD excluded significantly pre-term infants (below 34 weeks). Extreme prematurity or low birth weight is associated with subsequent lower mental development indices (Hack et al., 2005, Wood et al., 2000). The influence of fruit was greatest in late pre-term infants (at 34 weeks GA) with diminishing effects towards term. We could not determine the influence of prenatal fruit intake on infants born before 34 weeks GA. The mean calorie intake for the sample was consistent with recommendations for pregnant women from1800 calories in the first trimester to 2400 cal in the third trimester (National Institutes of Health and Services USDoHaH, 2016). However, we could not verify the accuracy of the nutrient or calorie intake as these are calculated value based on an analysis of the entire FFQ using the NDSR software (Dennis et al., 1980). Future studies are required to determine if gestational fruit consumption can mitigate the adverse neurobehavioral impact of prematurity. Related is identifying if fruit intake is differentially important during different trimesters.

The US Department of Agriculture (USDA) and US Department of Health and Human Services recommend 2 cups (3–4 servings (U.S. Department of Health and Human Services and U.S. Department of Agriculture, 2000)) of fruit per day for active women. One cup of fruit (U.S. Department of Health and Human Services and U.S. Department of Agriculture, 2005) (2 servings) consists of either 1 cup of raw or cooked fruit, 1 cup (8 oz) of 100% fruit juice, or a small whole fruit. The results of our study support an increased consumption of fruit (6–7 servings; 3 cups) for pregnant women. However, further research is required to examine the potential impact of increased fruit consumption on the development of gestational diabetes and the impact of gestational fruit consumption on other health outcomes including birth weight and infant behavior.

Increased gestational fruit consumption in healthy individuals is associated with improved cognitive development in children at 1 year of age in our population-based cohort. Fruit nutrient components (lycopene, fructose) were both associated with neurodevelopment. Validation in *Drosophila* helps control for potential participant bias and unmeasured confounders. Results of this study strongly support the consumption of fruit as part of the prenatal diet in the general population of pregnant women.

## Author Contributions

P.M. conceived the study, obtained funding for the study, wrote the first draft of the manuscript and drafted the final version of the manuscript. F.V.B. conceptualized, analyzed, prepared figures for the *Drosophila* experiments and co-wrote the manuscript. S.L. and C.R. performed the *Drosophila* memory experiments and prepared the figures. L.S. helped with manuscript development. A.L. performed statistical analysis, prepared figures and tables. J.P. and C.R. helped with designing and executing the CHILD neurodevelopmental testing. M.R.S., A.B.B., P.S. and, S.E.T. helped obtain funding, advised on the CHILD study design, and participated in data collection. L.L. N.W. C.D. and R.T.A. provided input into the *Drosophila* memory experiments. All authors provided critical comments on the manuscript content and approved the final version of the manuscript. This study was supported by CIHR, AllerGen Network of Centres of Excellence and the Women and Children's Health Research Institute (WCHRI).

## Competing Financial Interests

The authors declare no competing financial interests.

## Figures and Tables

**Fig. 1:**
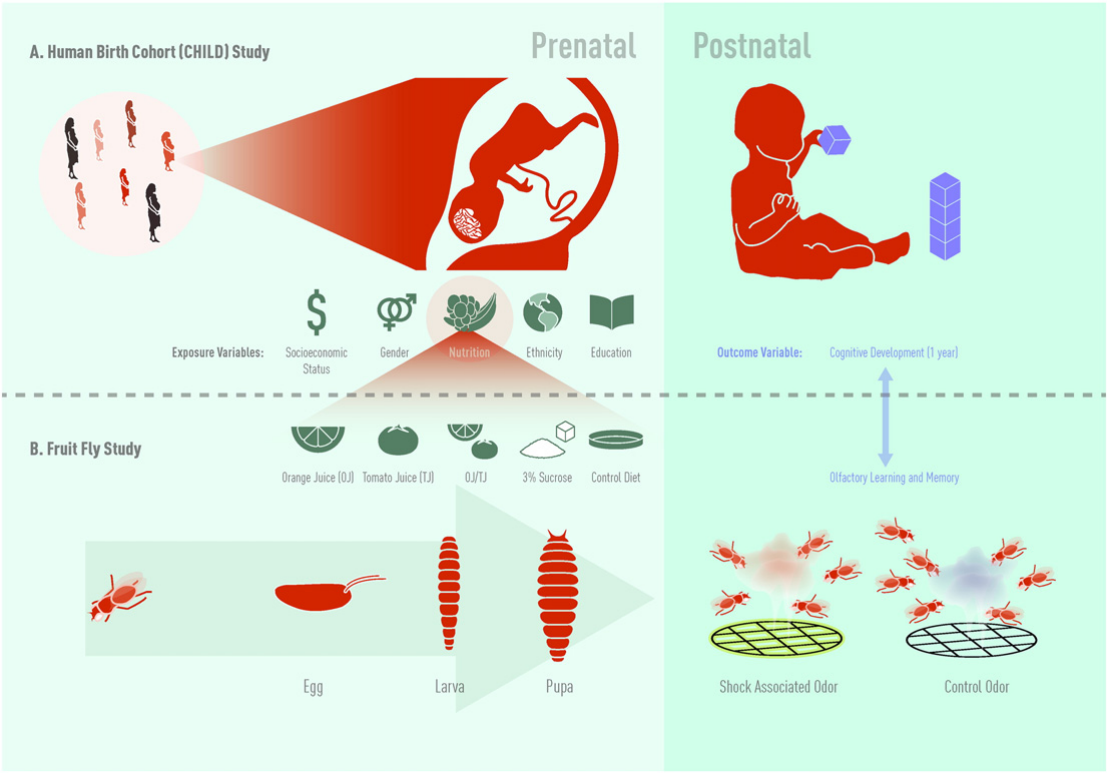
A) Study schema for the Canadian Healthy Infant Longitudinal Development (CHILD) study. B) Study schema for the Drosophila olfactory learning and memory study. Jason Everitt developed this figure and has authorized the use of this figure for use in this publication.

**Table 1:** Canadian Healthy Infant Longitudinal Development (CHILD) Edmonton sub-cohort study demographics.

	Cognitive			Adaptive		
Cognitive data present % (n/total)	Cognitive data absent % (n/total)	p-Value	Adaptive data present % (n/total)	Adaptive data absent % (n/total)	p-Value
*Categorical*						
Male	50.3 (346/688)	54.7 (64/117)	0.38	50.7 (334/659)	52.1 (76/146)	0.76
First-born	43.8 (300/685)	43.6 (51/117)	0.97	44.1 (289/656)	42.5 (62/146)	0.73
Married or common law	93.7 (611/652)	91.5 (97/106)	0.40	94.1 (587/624)	90.3 (121/134)	0.11
Income $60,000 or over	86.5 (572/661)	73.6 (67/91)	0.001	87.3 (556/637)	72.2 (83/115)	< 0.001
Mother attended post-secondary	92.6 (613/662)	81.4 (83/102)	< 0.001	93.2 (592/635)	80.6 (104/129)	< 0.001
Caucasian (child)	69.9 (467/668)	54.7 (58/106)	0.002	70.7 (454/642)	53.8 (71/132)	< 0.001
Smoking in the house	14.7 (86/586)	16.7 (3/18)	0.81	14.5 (84/579)	20.0 (5/25)	0.45
Children eating fruits at 6 months	54.3 (249/459)	66.7 (26/39)	0.13	54.5 (247/453)	62.2 (28/45)	0.32
Children eating fruits at 12 months	0.3 (2/592)	0 (0/16)	0.82	0.2 (1/585)	4.2 (1.24)	0.08

*Continuous*						
Gestational age during FFQ (weeks)	28.5 (27.9, 29.1) n = 602	28.2 (26.6, 29.8) n = 89	0.7	28.5 (27.90, 29.10) n = 579	28.4 (26.9, 30.0) n = 112	0.94
Gestational age (weeks)	39.5 (39.4, 39.6) n = 685	39.3 (39.09, 39.5) n = 117	0.27	39.5 (39.4, 39.6) n = 656	39.4 (39.15, 39.6) n = 146	0.36
Maternal age (years)	31.6 (31.2, 31.9) n = 688	29.8 (28.85, 30.64) n = 117	< 0.001	31.6 (31.2, 31.9) n = 659	30.1 (29.3, 30.9) n = 146	< 0.001
Healthy eating index	73.3 (72.7, 73.9) n = 607	71.0 (68.87, 73.2) n = 89	0.02	73.3 (72.7, 73.9) n = 584	71.5 (69.57, 73.5) n = 112	0.04
Fruit consumption per day (servings)	3.0 (2.8, 3.1) n = 605	3.5 (3.06, 3.97) n = 89	0.01	3.0 (2.8, 3.1) n = 582	3.4 (3.0, 3.8) n = 112	0.04
Breastfeeding duration (months)	7.8 (7.5, 8.1) n = 688	7.3 (6.7, 7.9) n = 117	0.17	7.9 (7.6, 8.2) n = 659	7.1 (6.6, 7.7) n = 146	0.03
Calories per day	2093 (2015, 2151) n = 605	2440 (2232, 2649) n = 89	0.001	2084 (2026, 2142) n = 582	2416 (2232, 2601) n = 112	0.04

**Table 2:** Multivariate regression analysis for cognitive development at 1 year of age. Column 1 uses gestational fruit intake as a continuous predictor. Column 2 categorizes gestational fruit consumptions into a dichotomous predictor above and below 7 servings of fruit per day. Column 3 replaces fruit consumption with specific fruit nutrients (e.g. lycopene) as a predictor variable. The complete regression is presented in Supplemental Table e5.

	Change in cognitive development composite score (95% CI)		
Fruit (continuous)	Fruit (categorical)	Nutrients
Child's gender (ref: male) female	0.33 (− 1.18, 1.84)	0.38 (− 1.13, 1.88)	0.41 (− 1.1, 1.92)
Mother's ethnicity (ref: Caucasian) Other	− 0.95 (− 2.83, 0.92)	− 0.98 (− 2.86, 0.9)	− 0.91 (− 2.79, 0.97)
Family income (ref: <$60,000) ≥ $60,000	0.23 (− 2.08, 2.55)	0.39 (− 1.93, 2.7)	0.3 (− 2.01, 2.62)
Mother's attended post-secondary school (ref: did not attended post-secondary school)	4.64⁎ (1.62, 7.65)	4.91⁎ (1.9, 7.93)	5.00⁎ (1.98, 8.01)
Daily prenatal fruit intake (per serving)	2.38⁎ (0.39, 4.37)		
Daily prenatal fruit intake ≥ 7 servings/day (ref: < 7 servings)		5.10⁎ (0.65, 9.55)	
Daily prenatal lycopene intake (mg)			0.14⁎ (0, 0.28)
Healthy eating index	− 0.05 (− 0.16, 0.06)	− 0.03 (− 0.13, 0.07)	− 0.02 (− 0.13, 0.08)
Post-natal fruit in infant diet at 6 months (ref: no) yes	0.98 (− 0.85, 2.81)	0.97 (− 0.86, 2.81)	0.97 (− 0.86, 2.81)
Maternal calcium consumption pre-pregnancy (ref: never) < 1/week	− 4.68⁎ (− 7.88,− 1.48)	− 4.51⁎ (− 7.7, − 1.31)	− 4.40 (− 7.6, − 1.2)
≥ 1/week	− 1.83 (− 3.86, 0.2)	− 2.13⁎ (− 4.15, − 0.1)	− 1.92 (− 3.95, 0.11)
Prenatal calcium intake (per mg)	0.0006 (− 0.0009, 0.0020)	0.0004 (− 0.0009, 0.0018)	0.0006 (− 0.0008, 0.00019)
Gestational age (GA; weeks)^a^	− 2.13⁎ (− 3.38, − 0.90)	0.86⁎ (0.25, 1.47)	0.87 (0.26, 1.48)
GA at time of FFQ completion	− 0.15⁎ (− 0.26, − 0.04)	− 0.15⁎ (− 0.26, − 0.04)	− 0.14⁎ (− 0.25, − 0.03)
Prenatal fruit intake and GA interaction	0.36 (0.00, 0.73)		
Gestational diabetes (GDM) yes	− 9.19⁎ (− 18.33, − 0.05)	− 9.85⁎ (− 18.98, − 0.71)	− 10.04⁎ (− 19.19, − 0.89)
GA and GDM interaction	2.04⁎ (0.07, 4.01)	2.15⁎ (0.18, 4.12)	2.13⁎ (0.15, 4.1)

⁎ p < 0.05.

a Centred at 34.29 weeks gestational age (34.29 — gestational age).

**Table 3:** Multivariate regression analysis for adaptive development at 1 year of age. Column 1 uses gestational fruit intake as a continuous predictor. Column 2 replaces fruit consumption with specific fruit nutrients (e.g. lycopene and fructose) as predictor variables. The complete regression is presented in Supplemental Table e6.

	Change in adaptive development composite score (95% CI)	
Fruit (continuous)	Nutrients
Child's gender (ref: male) female	1.36 (− 0.61, 3.33)	1.37 (− 0.59, 3.33)
Birth order (ref: first born) subsequent born	− 1.86 (− 3.94, 0.21)	− 1.69 (− 3.76, 0.38)
Gestational age (GA; weeks)^a^	0.98 (0.23, 1.74)	0.94 (0.18, 1.7)
Mother's ethnicity (ref: Caucasian) Other	− 1.00 (− 3.48, 1.48)	− 0.92 (− 3.39, 1.55)
Maternal age (years)	− 0.30 (− 0.54, − 0.05)	− 0.3 (− 0.54, − 0.05)
Family income (ref: <$60,000) ≥ $60,000	1.70 (− 1.46, 4.87)	1.72 (− 1.43, 4.88)
Mother's attended post-secondary school (ref: did not attended post-secondary)	− 1.94 (− 6.13, 2.26)	− 1.49 (− 5.67, 2.69)
Gestational diabetes (GDM) yes	− 1.16 (− 4.92, 2.6)	− 1.25 (− 5, 2.51)
Daily prenatal fruit intake (per serving)	0.67⁎ (0.07, 1.27)	
Daily prenatal lycopene intake (mg)		0.2 (0.01, 0.39)
Daily prenatal fructose intake (g)		0.06⁎ (0, 0.13)
Post-natal fruit in infant diet at 6 months (ref: no) yes	2.83⁎ (0.44, 5.21)	2.66⁎ (0.28, 5.03)
Healthy eating index	− 0.03 (− 0.17, 0.11)	0.01 (− 0.13, 0.14)
GA at time of FFQ completion	− 0.02 (− 0.16, 0.12)	0.01 (− 0.16, 0.13)

⁎ p < 0.05.

a Centered at 34.29 weeks gestational age.

## Appendix A Supplementary Data

Supplementary material. Image 2
