# Supplementary material for: Cognitive Enhancement in Infants Associated with Increased Maternal Fruit Intake During Pregnancy: Results from a Birth Cohort Study with Validation in an Animal Model
Source: eBioMedicine. 2016 Apr 22;8:331–40. doi: 10.1016/j.ebiom.2016.04.025 (PMC4919537; doi:10.1016/j.ebiom.2016.04.025)
Supplement: Supplementary file 1 — Supplementary material. [file mmc1.docx]

**Online Supplemental Material:**

**CHILD Study investigators contributors (alphabetical order):**

| **Author** | **Affiliation** |  | **Author** | **Affiliation** |
| --- | --- | --- | --- | --- |
| Anand SS | McMaster University |  | Allen R | Simon Fraser University |
| Becker AB, | University of Manitoba |  | Befus AD | University of Alberta |
| Brauer M | University of British Columbia |  | Brook JR | University of Toronto |
| Chen E | Northwestern University, Chicago |  | Cyr M | McMaster University |
| Daley D | University of British Columbia |  | Dell S | The Hospital for Sick Children |
| Denburg JA | McMaster University |  | Elliott S | University of Waterloo |
| Grasemann H | The Hospital for Sick Children |  | HayGlass K | University of Manitoba |
| Hegele R | The Hospital for Sick Children |  | Holness DL | University of Toronto |
| Lou WYW | University of Toronto |  | Kobor MS | University of British Columbia |
| Kollman TR | University of British Columbia |  | Kozyrskyj AL | University of Alberta |
| Laprise C | Université du Québec à Chicoutimi |  | Larché M | McMaster University |
| Macri J | McMaster University |  | Mandhane PJ | University of Alberta |
| Miller G | Northwestern University, Chicago |  | Moraes T | The Hospital for Sick Children |
| Paré PD | University of British Columbia |  | Ramsey C | University of Manitoba |
| Ratjen F | The Hospital for Sick Children |  | Sandford A | University of British Columbia |
| Scott JA | University of Toronto |  | Scott J | University of Toronto |
| Sears MR, (Director) | McMaster University |  | Silverman F | University of Toronto |
| Subbarao P  (co-Director) | The Hospital for Sick Children |  | Takaro T | Simon Fraser University |
| Tebbutt S | University of British Columbia |  | To T | The Hospital for Sick Children |
| Turvey SE | University of British Columbia |  |  |  |

**Online Supplemental Material: Tables and figures legend**

**Supplemental Figure e1: Fruit feeding does not affect sensory controls**. **A)** No significant changes were observed in shock sensation or **B)** olfactory avoidance of wild type flies fed prenatally with enriched fruit compared to regular diet. **C)** No significant changes were observed in shock sensation or **D)** olfactory avoidance of wild type flies fed with enriched fruit compared to regular diet as adult for 4 days. **E)** No significant changes were observed in shock sensation or **F)** olfactory avoidance of wild type or rutabaga mutant flies fed prenatally with enriched fruit compared to regular diet.

**Supplemental table e1:** Study inclusion and exclusion criteria for the CHILD study

**Supplemental table e2:** CHILD Edmonton study overview

**Supplemental Table e3a:** Univariate analysis (categorical predictors) - cognitive development at 1 year of age

**Supplemental Table e3b:** Univariate analysis (continuous predictors) - cognitive development at 1 year of age

**Supplemental table e4a:** univariate analysis (categorical variables) - adaptive development at 1 year of age

**Supplemental table e4b:** univariate analysis (continuous variables) - adaptive development at 1 year of age

**Supplemental table e5:** multivariate analysis - cognitive development at 1 year of age

**Supplemental table e6:** multivariate analysis - adaptive development at 1 year of age


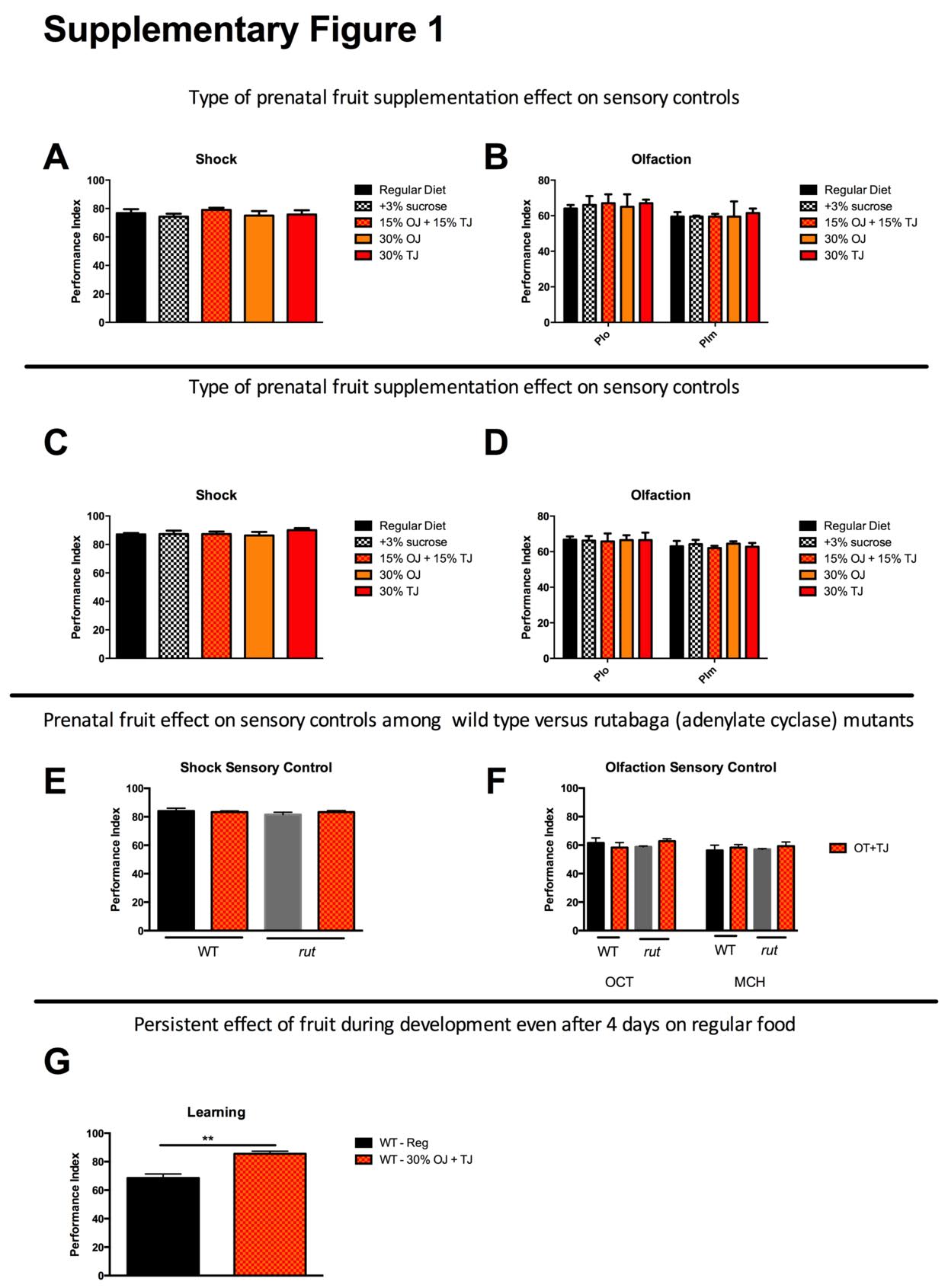


**Supplemental Table e1:** Study inclusion and exclusion criteria for the CHILD study

***Inclusion Criteria***

1. Pregnant women aged 18 years and older

2. Residence in reasonable proximity to the Stollery Children’s Hospital

3. Able to read and speak English

4. Willing to provide informed consent

5. Willing to consent to cord blood collection by the study

6. Planning to give birth at the recruitment centre

7. Infants born at or after 36 weeks

8. Must be able to provide a valid address and telephone number and names and phone numbers of two alternate contact individuals

***Exclusion Criteria***

1. Children with major congenital abnormalities or respiratory distress syndrom (RDS)

2. Infants born before 36 weeks gestation

3. Expectation of moving away from a recruitment area within 1 year

4. Children of multiple births

5. Children resulting from in-vitro fertilization

6. Children who do not spend at least 80% of nights in the index home

**Supplemental table e2:** Study overview for Edmonton sub-cohort of CHILD

|  |  | Prenatal | | Birth | Post-natal | | | |
| --- | --- | --- | --- | --- | --- | --- | --- | --- |
|  | Q: Questionnaire  P: Physiological Testing  B: Biological Sample  E: Environmental assessment  Sleep and neurobehavioral measures:  shaded grey | 18 to  36 wk  C  L  I  N  I  C | 36 wk  C  L  I  N  I  C | H  O  S  P  I  T  A  L | 3 mo  H  O  M  E | 6 mo  P  H  O  N  E | 9 mo  P  H  O  N  E | 1 yr  C  L  I  N  I  C |
| Mother | Q: Health and Medications | ✓ |  |  |  |  |  | ✓ |
|  | Q: Environment | ✓ |  |  | ✓ | ✓ |  | ✓ |
|  | Q: Nutrition | ✓ |  |  | ✓ |  |  | ✓ |
|  | Q: Activity | ✓ |  |  |  |  |  |  |
|  | Q: Stress | ✓ | ✓ |  |  |  |  | ✓ |
|  | Q: Socio-economic status | ✓ |  |  |  |  |  | ✓ |
|  | Q: Delivery |  |  | ✓ |  |  |  |  |
|  | Q: Sleep |  |  |  |  |  |  | ✓ |
|  | P: Skin Prick Testing |  |  |  |  |  |  | ✓ |
|  | B: Breast Milk |  |  |  | ✓ |  |  |  |
| Father | Q: Health | ✓ |  |  |  |  |  |  |
|  | Q: Environment | ✓ |  |  |  |  |  |  |
|  | Q: Socio-economic status | ✓ |  |  |  |  |  | ✓ |
|  | Q: Sleep | ✓ |  |  |  |  |  |  |
|  | P: Skin Prick Testing | ✓ |  |  |  |  |  |  |
| Infant | Q: Health and Medications |  |  |  | ✓ | ✓ | ✓ | ✓ |
|  | Q: Infection |  |  |  | ✓ | ✓ | ✓ | ✓ |
|  | Q: Activity |  |  |  | ✓ | ✓ |  | ✓ |
|  | Q: Nutrition |  |  |  | ✓ | ✓ |  | ✓ |
|  | Q: Pediatric Sleep Questionnaire |  |  |  | ✓ | ✓ | ✓ | ✓ |
|  | Q: Brief Infant Sleep Questionnaire |  |  |  | ✓ | ✓ | ✓ | ✓ |
|  | Q: BSID-III |  |  |  |  |  |  | ✓ |
|  | P: Polysomnography |  |  |  |  |  |  | ✓ |
|  | P: Skin Prick Testing |  |  |  |  |  |  | ✓ |
|  | P: Height and Weight |  |  | ✓ |  |  |  | ✓ |

BSID-III: Bayley scale of infant development version III

**Supplemental Table e3a:** Univariate analysis (categorical predictors) - cognitive development at 1 year of age

|  |  | % (n/total) | Average score (95% CI) | p-value |
| --- | --- | --- | --- | --- |
| Categorical |  |  |  |  |
| Child's gender | Male | 50.29 (346/688) | 109.49 (108.38,110.61) | reference |
|  | Female | 49.71 (342/688) | 110.32 (109.28,111.36) | 0.29 |
| Birth Order | First Born | 43.60 (300/688) | 109.47 (108.3,110.64) | reference |
|  | Subsequent | 55.96 (385/688) | 110.16 (109.15,111.17) | 0.38 |
|  | Missing | 0.44 (3/688) | 120 (108.66,131.34) | 0.08 |
| Marital Status | Married or Common Law | 88.81 (611/688) | 109.85 (109.03,110.66) | reference |
|  | Divorce or Separated/Single | 5.96 (41/688) | 110.24 (107.07,113.42) | 0.81 |
|  | Missing | 5.23 (36/688) | 110.42 (107.59,113.25) | 0.75 |
| Family Income | <$60,000 | 12.94 (89/688) | 108.6 (106.51,110.68) | reference |
|  | >=$60,000 | 83.14 (572/688) | 110.11 (109.28,110.94) | 0.19 |
|  | Missing | 3.92 (37/688) | 109.81 (105.08,114.54) | 0.59 |
| Child's Ethnicity | Caucasian | 67.88 (467/688) | 110.36 (109.43,111.28) | reference |
|  | Other | 29.22 (201/688) | 109.05 (107.64,110.47) | 0.13 |
|  | Missing | 2.91 (20/688) | 107.75 (103.28,112.22) | 0.26 |
| Mother's education | Did not attend post secondary | 7.12 (49/688) | 105.55 (102.55,108.55) | reference |
|  | Attended post secondary | 89.10 (613/688) | 110.27 (109.47,111.07) | 0.002 |
|  | Missing | 3.78 (26/688) | 109.42 (105.97,112.88) | 0.12 |
| Mother's ethnicity | Caucasian | 76.89 (529/688) | 110.19 (109.34,111.04) | reference |
|  | Other | 21.66 (149/688) | 108.72 (106.98,110.47) | 0.12 |
|  | Missing | 1.45 (10/688) | 112 (104.95,119.05) | 0.58 |
| Gestational diabetes | No | 91.28 (628/688) | 110 (109.2,110.79) | reference |
|  | Yes | 7.70 (53/688) | 108.21 (105.33,111.08) | 0.22 |
|  | Missing | 1.02 (7/688) | 114.29 (107.71,120.86) | 0.27 |
| Smoking in the house | No | 85.32 (500/688) | 110.17 (109.29,111.06) | reference |
|  | Yes | 14.68 (86/688) | 108.02 (106.01,110.04) | 0.07 |
|  | Missing | 14.83 (102/688) | 110.15 (107.94, 112.35) | 0.98 |
| Child eating fruit at 6 month | No | 30.52 (210/688) | 109.13 (107.77, 110.48) | reference |
|  | Yes | 36.19 (249/688) | 110.38 (109.05, 111.72) | 0.19 |
|  | Missing | 33.28 (229/688) | 110.09 (108.82, 111.35) | 0.33 |
| Prenatal daily fruit intake | 0-6 servings | 84.74 (583/688) | 110 (109.18,110.83) | reference |
|  | 7+ servings | 3.20 (22/688) | 116.14 (111.39,120.88) | 0.01 |
|  | Missing | 12.06 (83/688) | 107.53 (105.42,109.64) | 0.04 |
| Mother's calcium intake prior to pregnancy | Never | 71.08 (489/688) | 100.74 (109.83, 111.64) | reference |
|  | <1/week | 5.96 (41/688) | 106.46 (103.72, 109.21) | 0.01 |
|  | >=1/week | 17.15 (118/688) | 108.53 (106.65, 110.42) | 0.03 |
|  | Missing | 5.81 (40/688) | 107.25 (104.25, 110.25) | 0.04 |
| Father's education | Did not attend post-secondary | 15.8 (104/660) | 105.55 (102.55,108.55) | reference |
|  | Attended post-secondary | 84.2 (556/660) | 110.27 (109.47,111.07) | 0.83 |

**Supplemental Table e3b:** Univariate analysis (continuous predictors) - cognitive development at 1 year of age

|  |  | n | Co-efficient (95% CI) | p-value |
| --- | --- | --- | --- | --- |
| Continuous |  |  |  |  |
| Healthy Eating Index |  | 607 | 0.01 (0.09, 0.11) | 0.84 |
| Average fruit intake per week (servings) |  | 605 | 0.59 (0.15, 1.04) | 0.01 |
| Calcium (mg) |  | 605 | 0.001 (0.00, 0.002) | 0.15 |
| Fructose (g) |  | 605 | 0.05 (0.00, 0.09) | 0.03 |
| Glucose (g) |  | 605 | 0.05 (0.00, 0.10) | 0.05 |
| Inositol (g) |  | 605 | 4.05 (0.50, 7.60) | 0.03 |
| Lycopene (mg) |  | 605 | 0.19 (0.06, 0.33) | 0.01 |
| Sorbitol (g) |  | 605 | 1.82 (0.50, 3.14) | 0.01 |
| Vitamin C (mg) |  | 605 | 0.01 (0.00, 0.02) | 0.05 |
| Gestational age (weeks) |  | 685 | 1.14 (0.58, 1.70) | <0.001 |
| Breastfeeding duration (months) |  | 688 | 0.06 (-0.14, 0.26) | 0.56 |

**Supplemental table e4a:** Univariate analysis (categorical variables) - Adaptive development at 1 year of age

|  |  | % (n/total) | Average score (95% CI) | p-value |
| --- | --- | --- | --- | --- |
| Categorical |  |  |  |  |
| Child's gender | Male | 50.68 (334/659) | 99.49 (98.13, 100.86) | reference |
|  | Female | 49.32 (325/659) | 100.56 (99.13, 102.00) | 0.29 |
| Birth Order | First Born | 43.85 (289/659) | 101.64 (100.16, 103.12) | reference |
|  | Subsequent | 55.69 (367/659) | 98.80 (97.47, 100.12) | 0.01 |
|  | Missing | 0.46 (3/659) | 93.33 (75.98, 110.69) | 0.27 |
| Marital Status | Married or Common Law | 89.07 (587/659) | 100.11 (99.08, 101.13) | reference |
|  | Divorce or Separated/Single | 5.61 (37/659) | 99.22 (94.23, 104.20) | 0.69 |
|  | Missing | 5.31 (35/659) | 99.40 (94.29, 104.51) | 0.75 |
| Family Income | <$60,000 | 12.29 (81/659) | 99.93 (96.74, 103.11) | reference |
|  | >=$60,000 | 84.37 (556/659) | 100.13 (99.07, 101.18) | 0.90 |
|  | Missing | 3.34 (22/659) | 97.64 (92.09, 103.18) | 0.46 |
| Child's Ethnicity | Caucasian | 68.89 (454/659) | 99.83 (98.66, 101.01) | reference |
|  | Other | 28.53 (188/659) | 100.20 (98.31, 102.08) | 0.75 |
|  | Missing | 2.58 (17/659) | 103.00 (95.44, 110.56) | 0.32 |
| Mother's education | Did not attend post secondary | 6.53 (43/659) | 101.67 (98.07, 105.28) | reference |
|  | Attended post secondary | 89.83 (592/659) | 99.88 (98.83, 100.92) | 0.38 |
|  | Missing | 3.64 (24/659) | 100.58 (94.76, 106.41) | 0.74 |
| Mother's ethnicity | Caucasian | 78.15 (515/659) | 100.44 (99.32, 101.55) | reference |
|  | Other | 20.79 (137/659) | 98.61 (96.48, 100.73) | 0.14 |
|  | Missing | 1.06 (7/659) | 97.14 (81.53, 112.76) | 0.50 |
| Gestational diabetes | No | 91.05 (600/659) | 100.33 (99.31, 101.34) | reference |
|  | Yes | 7.89 (52/659) | 97.54 (93.53, 101.54) | 0.14 |
|  | Missing | 1.06 (7/659) | 92.14 (79.19, 105.10) | 0.10 |
| Smoking in the house | No | 75.11 (495/659) | 99.88 (98.76, 101.01) | reference |
|  | Yes | 12.75 (84/659) | 100.89 (98.06, 103.72) | 0.51 |
|  | Missing | 12.14 (80/659) | 99.94 (96.91, 102.96) | 0.97 |
| Child eating fruit at 6 month | No | 31.26 (206/659) | 98.33 (96.54, 100.13) | reference |
|  | Yes | 37.48 (247/659) | 101.59 (100.00, 103.18) | 0.01 |
|  | Missing | 31.26 (206/659) | 99.82 (98.06, 101.58) | 0.24 |
| Prenatal daily fruit intake | 0-6 servings | 85.28 (562/659) | 99.72 (98.65, 100.78) | reference |
|  | 7+ servings | 3.03 (20/659) | 102.05 (96.49, 107.61) | 0.43 |
|  | Missing | 11.68 (77/659) | 101.71 (98.69, 104.74) | 0.20 |

**Supplemental table e4b:** Univariate analysis (continuous variables) - Adaptive development at 1 year of age

|  |  | n | Co-efficient (95% CI) | p-value |
| --- | --- | --- | --- | --- |
| Continuous |  |  |  |  |
| Healthy Eating Index |  | 584 | 0.00 (-0.13, 0.13) | 1.00 |
| Average fruit intake per week (servings) |  | 582 | 0.64 (0.06, 1.21) | 0.03 |
| Average vegetable intake per week (servings) |  | 582 | 0.62 (-0.02, 1.26) | 0.06 |
| Fructose (g) |  | 582 | 0.09 (0.04, 0.15) | 0.002 |
| Glucose (g) |  | 582 | 0.10 (0.04, 0.16) | 0.002 |
| Inositol (g) |  | 582 | 5.73 (1.21, 10.24) | 0.01 |
| Lycopene (mg) |  | 582 | 0.27 (0.10, 0.45) | 0.002 |
| Total sugars (g) |  | 582 | 0.02 (0.00, 0.04) | 0.02 |
| Vitamin C (mg) |  | 582 | 0.01 (0.00, 0.02) | 0.04 |
| Water (g) |  | 582 | 0.001 (0.00, 0.002) | 0.01 |
| Gestational age (weeks) |  | 656 | 1.12 (0.38, 1.85) | 0.003 |
| Maternal age (years) |  | 659 | -0.39 (-0.61, -0.16) | 0.001 |
| Breastfeeding duration (months) |  | 659 | -0.05 (-0.30, 0.21) | 0.72 |

**Supplement Table e5:** Multivariate regression analysis for cognitive development at 1 year of age. Column 1 uses gestational fruit intake as a continuous predictor. Column 2 categorizes gestational fruit consumptions into a dichotomous predictor above and below 7 servings of fruit per day. Column 3 replaces fruit consumption with specific fruit nutrients (e.g. lycopene) as a predictor variable.

|  | Fruit (continuous) | Fruit (categorical) | Nutrients |
| --- | --- | --- | --- |
|  | Change in cognitive development composite score  (95% CI) | Change in cognitive development composite score  (95% CI) | Change in cognitive development composite score  (95% CI) |
| Child's gender (ref: Male) |  |  |  |
| Female | 0.33  (-1.18, 1.84) | 0.38  (-1.13, 1.88) | 0.41  (-1.1, 1.92) |
| Mother's ethnicity (ref: Caucasian) |  |  |  |
| Other | -0.95  (-2.83, 0.92) | -0.98  (-2.86, 0.9) | -0.91  (-2.79, 0.97) |
| Missing | 4.42  (-2.49, 11.33) | 4.52  (-2.39, 11.43) | 4.65  (-2.27, 11.57) |
| Family Income (ref: <$60,000) |  |  |  |
| >=$60,000 | 0.23  (-2.08, 2.55) | 0.39  (-1.93, 2.7) | 0.3  (-2.01, 2.62) |
| Missing | 0.55  (-3.85, 4.94) | 0.25  (-4.15, 4.66) | 0.28  (-4.13, 4.69) |
| Mother's education (ref: Did not attended post secondary) |  |  |  |
| Attended post secondary | 4.64*  (1.62, 7.65) | 4.91*  (1.9, 7.93) | 5.00*  (1.98, 8.01) |
| Missing | 6.37*  (0.51, 12.23) | 6.69*  (0.83, 12.55) | 6.61*  (0.75, 12.48) |
| Gestational diabetes |  |  |  |
| Yes | -9.19*  (-18.33, -0.05) | -9.85*  (-18.98, -0.71) | -10.04*  (-19.19, -0.89) |
| Missing | 3.91  (-17.26, 25.08) | 6.05  (-15.05, 27.15) | 3.66  (-17.62, 24.94) |
| Daily prenatal fruit intake (per serving) | 2.38*  (0.39, 4.37) |  |  |
| Missing | -2.99  (-16.97, 10.98) |  |  |
| Daily prenatal fruit intake (ref: <7 servings) |  |  |  |
| =>7 servings |  | 5.10*  (0.65, 9.55) |  |
| Missing |  | 4.20  (-9.75, 18.15) |  |
| Daily prenatal lycopene intake (mg) |  |  | 0.14*  (0, 0.28) |
| Missing |  |  | -4.06  (-18.02, 9.9) |
| Post-natal fruit in infant diet at 6 months of age (ref: No) |  |  |  |
| Yes | 0.98  (-0.85, 2.81) | 0.97  (-0.86, 2.81) | 0.97  (-0.86, 2.81) |
| Missing | 1.73  (-0.23, 3.69) | 1.72  (-0.25, 3.68) | 1.65  (-0.31, 3.62) |
| Mother's calcium intake prior to pregnancy (ref: Never) |  |  |  |
| <1/week | -4.68*  (-7.88, -1.48) | -4.51*  (-7.7, -1.31) | -4.4  (-7.6, -1.2) |
| >=1/week | -1.83  (-3.86, 0.2) | -2.13*  (-4.15, -0.1) | -1.92  (-3.95, 0.11) |
| Missing | -3.7  (-8.55, 1.15) | -3.59  (-8.44, 1.26) | -3.68  (-8.54, 1.17) |
| Prenatal calcium intake (per mg) | 0.0006  (-0.0009, 0.0020) | 0.0004  (-0.0009, 0.0018) | 0.0006  (-0.0008, 0.00019) |
| Gestational age (weeks)** | -2.13*  (-3.38, -0.90) | 0.86*  (0.25, 1.47) | 0.87  (0.26, 1.48) |
| Missing | -7.02  (-22.12, 8.08) | -8.81  (-24.95, 7.34) | -8.58  (-24.74, 7.58) |
| Gestational age at time of FFQ completion | -0.15*  (-0.26, -0.04) | -0.15*  (-0.26, -0.04) | -0.14*  (-0.25, -0.03) |
| Healthy Eating Index | -0.05  (-0.16, 0.06) | -0.03  (-0.13, 0.07) | -0.02  (-0.13, 0.08) |
| Missing | 6.4  (-7.81, 20.61) | 6.81  (-7.4, 21.02) | 6.77  (-7.45, 21) |
| Prenatal fruit intake and gestational age interaction | 0.36  (0.00, 0.73) |  |  |
| Gestational diabetes and gestational age interaction |  |  |  |
| Yes | 2.04*  (0.07, 4.01) | 2.15*  (0.18, 4.12) | 2.13*  (0.15, 4.1) |
| Missing | -1.05  (-5.8, 3.7) | -1.42  (-6.15, 3.32) | -0.99  (-5.75, 3.78) |
| Constant | 107.09  (87.2, 126.98) | 108.19  (84.11, 132.27) | 110.41  (91.28, 129.54) |

*p<0.05

**Centred at 34.29 weeks gestational age (34.29 – gestational age)

**Supplemental Table e6:** Multivariate regression analysis for adaptive development at 1 year of age. Column 1 uses gestational fruit intake as a continuous predictor. Column 2 replaces fruit consumption with specific fruit nutrients (e.g. lycopene and fructose) as predictor variables.

|  | Fruit (continuous) | Nutrients |
| --- | --- | --- |
|  | Change in adaptive development composite score  (95% CI) | Change in adaptive development composite score  (95% CI) |
| Child's gender (ref: Male) |  |  |
| Female | 1.36 (-0.61, 3.33) | 1.37 (-0.59, 3.33) |
| Birth order (ref: First Born) |  |  |
| Subsequent Born | -1.86 (-3.94, 0.21) | -1.69 (-3.76, 0.38) |
| Missing | -0.5 (-20.03, 19.03) | 0.49 (-18.95, 19.93) |
| Mother's ethnicity (ref: Caucasian) |  |  |
| Other | -1.00 (-3.48, 1.48) | -0.92 (-3.39, 1.55) |
| Missing | -4.30 (-14.74, 6.15) | -4.03 (-14.42, 6.35) |
| Family Income (ref: <$60,000) |  |  |
| >=$60,000 | 1.70 (-1.46, 4.87) | 1.72 (-1.43, 4.88) |
| Missing | -0.91 (-7.07, 5.26) | -1.6 (-7.75, 4.54) |
| Mother's education (ref: Did not attended post secondary) |  |  |
| Attended post secondary | -1.94 (-6.13, 2.26) | -1.49 (-5.67, 2.69) |
| Missing | -0.91 (-7.73, 5.91) | -0.85 (-7.63, 5.94) |
| Gestational diabetes |  |  |
| Yes | -1.16 (-4.92, 2.6) | -1.25 (-5, 2.51) |
| Missing | -7.39 (-19.99, 5.22) | -8.5 (-21.07, 4.07) |
| Daily prenatal fruit intake (per serving) | 0.67* (0.07, 1.27) |  |
| Missing | -17.57 (-35.54, 0.39) |  |
| Daily prenatal lycopene intake (mg) |  | 0.2 (0.01, 0.39) |
| Missing |  | -17.56 (-35.43, 0.31) |
| Daily prenatal fructose intake (g) |  | 0.06* (0, 0.13) |
| Post-natal fruit in infant diet at 6 months of age  (ref: No) |  |  |
| Yes | 2.83* (0.44, 5.21) | 2.66* (0.28, 5.03) |
| Missing | 1.27 (-1.34, 3.87) | 1.01 (-1.59, 3.61) |
| Healthy Eating Index | -0.03 (-0.17, 0.11) | 0.01 (-0.13, 0.14) |
| Missing | 14.77 (-3.48, 33.02) | 14.68 (-3.47, 32.83) |
| Gestational age at time of FFQ completion | -0.02 (-0.16, 0.12) | -0.01 (-0.16, 0.13) |
| Gestational age (weeks)** | 0.98 (0.23, 1.74) | 0.94 (0.18, 1.7) |
| Maternal age (years) | -0.30 (-0.54, -0.05) | -0.3 (-0.54, -0.05) |
| Constant | 107.37 (93.92, 120.82) | 102.55 (89.22, 115.88) |

*p<0.05

**centred at 34.29 weeks gestational age
